# To evaluate the efficacy and safety of PLC in perimenopausal or postmenopausal women with Hot Flashes: study protocol for a randomized controlled trial

**DOI:** 10.1186/s13063-019-3482-5

**Published:** 2019-07-10

**Authors:** Su-Ji Choi, Dong-Il Kim

**Affiliations:** Department of Obstetrics & Gynecology, College of Korean Medicine, Dongguk University Korean Medicine Hospital, 27 Dongguk-ro, Ilsandong-gu, Goyang-si, Gyeonggi-do 10326 Republic of Korea

**Keywords:** Menopause, Postmenopausal syndrome, Hot flashes, Pharmacoacupuncture, Hominis placenta, Pharmacopuncture, Herbal acupuncture, Aqua-acupuncture, Climacteric, Hot flush

## Abstract

**Background:**

Hot flashes are the most frequent symptoms of the menopause, with 10–20% of all postmenopausal women reporting nearly intolerable occurrences. Although pharmacopuncture with hominis placenta extract is one of the new acupuncture therapies popular in East Asian medicine with a known efficacy in treating facial flushing, there has been little research on the efficacy and safety of this extract. This study, therefore, aims to evaluate the efficacy and safety of pharmacopuncture with hominis placenta extract (PLC) compared to injections of normal saline, in perimenopausal and postmenopausal women in Korea.

**Methods/design:**

This study is a randomized placebo-controlled single-blind multi-center parallel-design trial. In total, 128 perimenopausal or postmenopausal women who meet the inclusion criteria will be recruited. The treatment group will receive PLC pharmacopuncture twice a week, for a total of 18 sessions over 9 weeks. The control group will receive injections of normal saline at the same acupoints during the same period. The post-treatment follow-up assessment will occur 4 weeks after the participant has completed the treatment.

**Discussion:**

We believe that this trial will provide evidence for the efficacy and safety of PLC pharmacopuncture as a treatment for hot flashes in perimenopausal and postmenopausal women.

**Trial registration:**

Clinical Research Information Service (CRIS), Republic of Korea, ID: KCT0003533, Registered on 20 February 2019.

**Electronic supplementary material:**

The online version of this article (10.1186/s13063-019-3482-5) contains supplementary material, which is available to authorized users.

## Background

Today, women live more than a third of their lives in the perimenopausal or postmenopausal stage. Hot flashes affect their quality of life, and are usually the most common symptom, as they are experienced by 75% of women [1]. Symptoms can last from 6 months to as long as 10 years [2]. Symptoms associated with hot flashes include a feeling of intense heat, sweating, weakness, changes in heart rate, panic disorder, and anger. Repeated night hot flashes can lead to insomnia, which eventually results in difficulty in concentrating, fatigue, and cognitive impairment [3].

Although hormone replacement therapy is the most effective and popular treatment for hot flashes [4, 5], it is not administered to many women due to its adverse side effects and potential risks [6]. Recent research suggest that hormone replacement therapy increases the risk of coronary heart disease, stroke, and breast and endometrial cancer, and this has consequently resulted in increasing interest in non-hormonal therapies [7–10]. Interest has increased in phytoestrogen therapy, but its effect on menopausal symptoms remains unclear [4]. Serotonin reuptake inhibitors are effective for hot flashes but are ineffective for other menopausal symptoms [7]. Due to the varied limitations and side effects of existing treatments, research into therapies for hot flashes in complementary alternative medicine and traditional Korean medicine (including acupuncture) is receiving greater attention [11–15].

A number of clinical studies have reported that acupuncture reduces the symptoms of hot flashes. Pharmacopuncture is a combination of acupuncture and medication, and is a new therapy in which a purified herbal medicine is injected at an acupoint. It is widely used in China and Korea, and is also known as acupoint injection, herbal acupuncture, aqua acupuncture, and aquapuncture [16]. With its increasing use, various studies on pharmacopuncture have been initiated and are ongoing. However, although pharmacopuncture is used for various perimenopausal or postmenopausal symptoms, it has been the subject of very little clinical research. According to a systematic review in 2009 of randomized control trials in Korean pharmaceutical research by Park [17], the majority of such control trials on pharmacopuncture looked at musculoskeletal disorders. Thus, currently there is insufficient evidence for the use of pharmacopuncture to treat gynecological disorders such as postmenopausal syndrome.

Pharmacopuncture with hominis placenta is a widely used therapy for gynecological disorders. Hominis placenta is an extract of human placenta comprising various cell proliferators, blood clotting factors, and hormones and their precursors, such as gonadotropin, prolactin, thyroid-stimulating hormone, steroid hormones, prostaglandin, lysozyme, kininase, histaminase, erythropoietin, phospholipids, and polysaccharides [18–20]. In fact, this medicinal material has been widely used for a long time in the clinical practice of Korean gynecology, and is generally prescribed as an oral medication or pharmacopuncture [21]. Moreover, hominis placenta has mainly been used for chronic consumptive diseases or weak conditions, infertility, sexual dysfunction, and menopausal disorders. Its clinical application in pharmacopuncture is well established for infertility, menopausal disorders, freckles [22], menstrual pain [23–25], facial paralysis [26], and postpartum diseases [27].

The clinical trial drug, PLC, is manufactured from Drug Master File (DMF)-registered raw materials (hominis placenta extract) and is based on a Unicenta injection manufactured by the drug company Unimed. The hominis placenta extract has recognized efficacy in anti-oxidation, wound recovery, and anti-inflammation, and for increasing the metabolism of tissue cells [28–31]. Considering that hominis placenta pharmacopuncture is widely used to treat hot flashes in clinical practice, we are running this clinical trial using the readily available quality-controlled PLC to verify its efficacy and safety. In addition, doctors and Korean medicine doctors will work together on this clinical trial using PLC manufactured in a similar way to the licensed Unicenta injections.

## Methods/design

### Study aims

The aim of the study is to evaluate the efficacy and safety of PLC used to treat hot flashes and other symptoms of perimenopausal or postmenopausal women.

### Design and setting

**Fig. 1:**
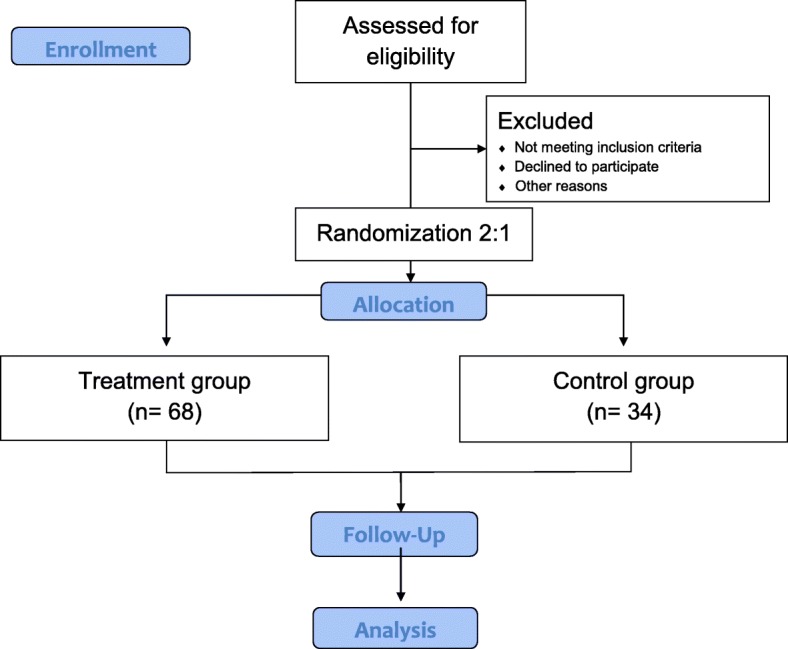
Participant flow diagram

**Fig. 2:**
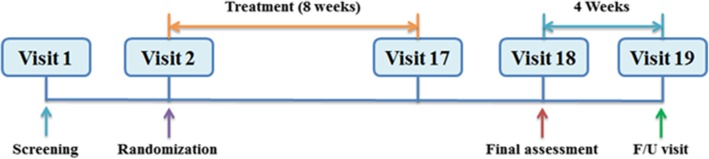
Study time line

This will be a randomized placebo-controlled single-blind multi-center parallel-design clinical trial. Participants will be enrolled from three hospitals: Ilsan Korean Medicine Hospital of Dongguk University, Gwangju Korean Medicine Hospital of Wonkwang University, and Dunsan Korean Medicine Hospital of Daejeon University. The study process is illustrated in Fig. 1. In accordance with the protocol, all subjects who sign the informed consent will undergo a 2-week screening period before commencing treatment. Subjects who complete the screening period and meet the selection criteria will be randomly assigned to one of two groups for 8 weeks: the treatment group, who will receive the PLC pharmacopuncture treatment, and the placebo group, who will receive injections of normal saline (NS). The relevant treatment material will be administered twice a week for 8 weeks (a total of 16 injections). From the second visit onwards, all subjects will receive a hot flash symptom diary, which they will be asked to bring to visits 4, 6, 8, 10, 12, 14, 16, and 18. The Menopause Rating Scale (MRS) will be assessed at visits 2, 10, and 18 for both groups. The post-treatment follow-up assessment will occur at visit 19, when the symptom diary and MRS questionnaires maintained by the subjects will be assessed for both groups (Figs. 1 and 2).

### Recruitment

Voluntary participants will be recruited form three centers: Ilsan Korean Medicine Hospital of Dongguk University, Dunsan Korean Medicine Hospital of Daejeon University, and Gwangju Korean Medicine Hospital of Wonkwang University. Recruitment will be through advertisements in outdoor areas, newspapers as well as on hospital websites.

### Eligibility criteria

#### Inclusion criteria

1. Women between 45 and 60 years of age
2. Perimenopausal or postmenopausal women experiencing hot flashes
3. Perimenopausal women should meet one of the following criteria:
  i. have a history of amenorrhea for the last 3–11 months from the consent day (visit 1)
  ii. have suffered irregular menstrual periods in the past 12 months from the consent day (visit 1)
4. Postmenopausal women should meet one of the following criteria:
  i. have a history of spontaneous amenorrhea for the last 12 months
  ii. have 6 months of spontaneous amenorrhea with serum follicle-stimulating hormone levels greater than 40 mIU/mL
  iii. have had a postsurgical bilateral oophorectomy with or without hysterectomy at least 6 weeks prior to the consent day
  iv. have had a hysterectomy
5. Women having an average daily hot flash score of 10 or higher for 1 week before visit 1
6. Subjects who agree to this clinical study after sufficient explanation

#### Exclusion criteria

1. Women diagnosed with psychogenic menopausal symptoms
2. Women suspected of having an organic disease
3. Women with severe complications of the heart, liver, kidney, or other organs
4. Women with a history of malignant tumors
5. Women with thyroid disease or abnormal thyroid function
6. Women with liver or kidney dysfunction, indicated by values of sGOT, sGPT, bilirubin, or serum creatinine over twice the normal level
7. Women who may have difficulties in participating in this trial due to any of the following diseases: uncontrolled hypertension, diabetes with complications or under control with insulin injections and pumps, thyroid disease including those taking medication for thyroid disease, acute hepatitis or hepatocirrhosis, severe hyperlipidemia, neuropathic disorders, severe cardiovascular disorders, tuberculosis, and other infectious diseases
8. Women with a history of hypersensitivity to hominis placenta or other drugs and food, or any other allergic disease that requires treatment
9. Women who have been administered hormones or hormone analogues, except estrogen or progestin, within the past 1 month
10. Women who have participated in another clinical trial within the past 3 months
11. Women who do not complete the required washout period for previous medication, such as Korean medicine treatment or other complementary medicine, that may affect perimenopausal or postmenopausal symptoms (Table 1)
12. Women who were previously taking estrogen or estrogen/progestin (except those who complete the washout period)
13. Women who have taken antidepressants in the last month
14. Women with any illness that is determined incompatible with the acupuncture treatment
15. Women with inflammation or a scar in the treatment area
16. Night-shift workers
17. Women who are unable to fill out the study documents
18. Any woman whom a researcher deems to be unsuitable for this trial

**Table 1:** Washout periods

Therapy	Washout period
Hormone agents vaginally (e.g. ring, cream, gel, etc.)	≥ 1 week
Transdermal agents containing estrogen-only or estrogen/progestin	≥ 4 weeks
Oral estrogen or progestin	≥ 8 weeks
Intrauterine progestin	≥ 8 weeks
Progestin implants or a single estrogen injection	≥ 12 weeks
Estrogen pellets or progestin injections	≥ 24 weeks
Acupuncture or moxibustion	≥ 1 week
Herbal medication or pharmacopuncture	≥ 3 weeks

### Interventions

For all subjects in the pharmacopuncture group, 0.5 cc of PLC will be injected twice weekly at four acupoints (CV4, CV6, and bilateral Ex-BB1) using a 1-cc 30-gauge disposable syringe. Needle insertion will be to a depth of up to 8 mm. The control group will be injected with NS at the same points using the same technique. After the procedure, participants will receive infrared irradiation at a distance of 30 to 60 cm for 20 min so that they feel warmth in their lower abdomen. No treatments other than the prescribed treatment should be received during the test period. The pharmacopuncture treatment will be administered by an experienced Korean medicine doctor who has had at least 6 years of regular Korean medicine university education and is licensed by the Korean Ministry of Health and Welfare.

The drug for this clinical trial, PLC, is an extract of hominis placenta and will be manufactured by pharmaceutical company Unimed (Seoul, Republic of Korea). Hominis placenta is isolated from healthy pregnant women after delivery, and will be subjected to sterilization and hydrolysis extraction procedures. PLC is manufactured in accordance with Korean good manufacturing practice. The NS for the control group consists of sodium chloride 180 mg/20 ml (0.9%) and will be procured from Daihan Pharm Co. (Seoul, Republic of Korea). A researcher will label the two test materials as PLC and NS.

### Randomization

Eligible participants will be randomized into one of the two groups via stratified block randomization at the hospital where they first come into contact with the research coordinators. The stratification factor is the hospital. A separate randomization sequence will be generated by computer at the central coordinating center by an independent statistician. The randomization codes will be sealed in opaque envelopes. Randomization plans will be kept confidential, except for emergencies. In the event of adverse events and emergencies related to the safety of the subjects, the test conductor in charge can identify the assigned treatment group of the subject through the statistician, who will maintain the random assignment table.

### Blinding

To eliminate differences between the PLC group and control group and to maintain the blinding, the NS will be administered in an identical way as the PLC. According to Yoon et al.’s study published in 2004 [32], NS is suitable as a control intervention for hominis placenta pharmacopuncture since the acupuncture sensation after injection is like that of the medication. The same amount of PLC or NS will be injected by the physician, at the same acupoints using the same gauge of syringe, so that participants do not feel any difference. Due to differences in the color of the PLC and the saline solution, the syringes will be covered with translucent tape to eliminate bias by the subjects.

### Outcome measures

#### Primary outcome: hot flash score

The primary outcome will be the mean change in the hot flash score from baseline to 8 weeks. The hot flash score for each day will be calculated as the number of hot flashes multiplied by the average severity of the hot flashes on that day. Participants will record the number of hot flashes in a daily diary during the trial. Flashes will be scored by severity on a scale from 0 to 4 (normal 0, mild 1, moderate 2, severe 3, and very severe 4) [33]. Since the hot flash score does not have a highest value, the residual hot flash score will be used for all statistical analyses. The hot flash score measured at the baseline will be normalized to 100, and the residual hot flash score will be calculated as a percentage of the baseline score.

#### Secondary outcomes

The secondary outcomes will be the mean changes in MRS, follicle-stimulating hormone levels, and estradiol levels, from baseline to 8 weeks. The MRS questionnaire is a self-administered instrument that has been widely used and validated. It has been used to assess the severity of menopausal symptoms in numerous clinical trials and epidemiological studies, and in research on the etiology of menopausal symptoms. The MRS questionnaire considers 11 symptoms, with each symptom being scored on a scale from 0 (no complaints) to 4 (very severe symptoms). The follicle-stimulating hormone and estradiol levels will be evaluated by blood tests on visits 1 and 18.

### Sample size

All analyses will be undertaken by a professional statistician. Because no prior research using hominis placenta pharmacopuncture to treat hot flashes has been published, we will reference studies that have used acupuncture alone [34] or human placenta extract alone [35, 36] to alleviate hot flashes. The standard deviation was conservatively assumed based on this previous research [34–36]. The population mean of the control group was estimated using the change from the baseline in studies of acupuncture, and the population mean of the treatment group was estimated by assuming that pharmacopuncture will be more effective than acupuncture alone. Hence, the effect value was calculated by combining estimates from studies of pure acupuncture with the weighted average from studies of herbal medications.

A minimum of 68 women in the treatment group and 34 women in the control group will be necessary to identify a 20% difference with a power of 80% and α = 0.05. Assuming a total withdrawal and dropout rate of 20%, we estimate that a total sample size of 128 subjects (85 in the treatment group and 43 in the control group) is required.

### Study visit and data collection

The schedule of follow-up visits is shown in Table 2.

**Table 2:** Schedule of follow-up visits

Period	Screening	Treatment																	Follow-up
Visit	1	2	3	4	5	6	7	8	9	10	11	12	13	14	15	16	17	18	19
Week	0	1		2		3		4		5		6		7		8		9	13
Enrollment:																			
Informed consent	×																		
Demographic characteristics	×																		
Medical history	×																		
Vital signs	×	×	×	×	×	×	×	×	×	×	×	×	×	×	×	×	×	×	×
Physical examination	×																	×	
Lab test	×																	×	
Inclusion/exclusion criteria	×																		
Randomization		×																	
Intervention:																			
Pharmacopuncture group		×	×	×	×	×	×	×	×	×	×	×	×	×	×	×	×	×	
Control group		×	×	×	×	×	×	×	×	×	×	×	×	×	×	×	×	×	
Assessment:				×		×		×		×		×							
Hot Flash Score	×	×		×		×		×		×		×		×		×		×	×
Menopause Rating Scale		×								×								×	×
Adverse events		×	×	×	×	×	×	×	×	×	×	×	×	×	×	×	×	×	×

### Statistical analysis

The analysis will primarily be a full analysis. In addition, a per-protocol analysis will be undertaken. The full analysis will include all participants who have completed at least one PLC or NS treatment session and whose primary outcome has been evaluated at least once. The per-protocol analysis will include only those patients who have completed at least 80% of the treatment sessions and for whom there are no serious violations of the protocol. For the safety assessment, we will analyze a safety group, which will include all participants who have received at least one session of PLC or NS treatment. Important variables that may affect the final evaluation will be considered in the analysis.

### Missing data

If data are missing for some time point or the participant has been lost to follow-up, a last observation carried forward analysis will be performed, as if it were obtained at that time. The last observation carried forward method will be applied to all efficacy assessment analyses. However, for laboratory test results, the analysis will be performed using only the available data sets.

### Additional analysis

#### Safety evaluation

At each visit, all subjective and objective discomforts will be collected through interviews, and these will be recorded on the case report form. The overall rate of adverse events will be calculated and analyzed. The rate of adverse events for each group will be compared using a chi-squared test or Fisher’s exact test. If it is necessary to control critical variables that may affect the final assessment, a layering analysis will be applied (e.g., the Cochran–Mantel–Haenszel method).

### Quality assurance

Quality standards will be set to ensure that patients are treated in accordance with this protocol. All practitioners will be informed of all details pertaining to this trial. Treatments will be performed by a Korean medicine doctor having extensive clinical experience in acupuncture and pharmacopuncture. Specifically, they will be graduates of a 6-year full-time course in Korean medicine taught as a college program. They must be certified by the Korean Ministry of Health and Welfare as a Korean medicine doctor and have more than 1 year of postgraduate clinical training in a Korean medicine hospital.

The contract research organization retains responsibility for quality control, and shall meet regularly throughout the study period. The contract research organization will frequently monitor all source documents and case report forms to ensure the quality and reliability of the clinical test results. Monitoring will be conducted in accordance with the clinical test management standards and relevant regulations. The study will be executed in accordance with the Helsinki declaration and good clinical practice requirement.

### Ethics

This study protocol has been approved by the Korean Ministry of Food and Drug Safety (approval 31743). Additionally, the trial has been authorized by the institutional review boards of Ilsan Korean Medicine Hospital of Dongguk University (approval DUIOH 2018–07–003-002), Dunsan Korean Medicine Hospital of Daejeon University (approval DJDSKH-18-DR-15), and Gwangju Korean Medicine Hospital of Wonkwang University (approval 2018/14). All participants must provide informed consent before enrollment. This trial is registered with the Korean Clinical Trial Registry (CRIS), Republic of Korea.

## Discussion

Due to the limited number of high-quality randomized controlled trials, there is no reliable evidence for the safety and efficacy of pharmacopuncture [37]. Thus, this is a randomized placebo-controlled single-blind multi-center parallel clinical study, designed to evaluate the efficacy and safety of hominis placenta pharmacopuncture in the treatment of perimenopausal or postmenopausal women experiencing hot flashes.

Based on the theory of Korean medicine, pharmacopuncture is a therapeutic method to cure diseases by injecting a pharmacopuncture solution at relevant acupoints or pain points. It is a new form of acupuncture treatment combining acupuncture and herbal medicine. In acupuncture, there is physical stimulation of associated meridians and acupoints, and pharmacopuncture adds chemical stimulation to acupuncture. Since herbal extracts can be absorbed directly without passing through the gastrointestinal tract, the effects of pharmacopuncture are expected to be realized more quickly than oral herbal medicine [38].

Hot flashes are the most common symptoms related to the menopausal transition, and the primary reason that women seek medical treatment for menopausal symptoms [36]. Hot flashes are the second most worrisome menopausal symptom after weight gain [39]. Hot flashes are associated with a decreased quality of life and sleep disorders, and may play a role in influencing both the intensity and prevalence of other symptoms experienced during the menopause [40, 41].

In a systematic review of pharmacopuncture in Korea, most published papers were on hominis placenta pharmacopuncture [37]. This extract is reported to be efficacious for leg spasticity [42], dyspepsia [43], Bell’s palsy [44], osteoarthritis [45], dysmenorrhea [24, 25], and postpartum symptoms [27]. Hominis placenta is widely taken by perimenopausal or postmenopausal women as an oral medication or via pharmacopuncture in Korean medicine. However, there have been no studies on its use for the climacteric syndrome.

Western medicine has associated climacteric symptoms with the reduced function of the hypothalamic-pituitary-gonadal axis. Conversely, traditional Korean medicine frequently attributes renal deficiency as the underlying mechanism for climacteric symptoms. The normal functions of the kidneys in Korean medicine include thermoregulation, sexuality, and water homeostasis. During the menopausal transition, kidney function may decline with increasing age. In Korean medicine, many menopausal symptoms, such as hot flashes, dry mucosa, sleep disorders, and recurrent urinary tract infections, are regarded as symptoms of a deficiency of yin in the kidneys. Yin is identified with receiving and regenerative elements. The hominis placenta is known to have the power to replenish the yin in the kidneys [46].

Despite the wide use of pharmacopuncture in Korean medicine, there are several issues with treatment. First, the efficacy of hominis placenta pharmacopuncture for the treatment of climacteric syndrome needs to be evaluated in large well-designed clinical trials. However, objectification and standardization of the medication is the first step in addressing the numerous problems inherent in operating a clinical trial on hominis placenta pharmacopuncture. We standardized the medication by using PLC manufactured following the guidelines for the Unicenta injection, after obtaining approval from the Korean Ministry of Food and Drug Administration. Unicenta is a commercially available injection. Published results of clinical trials in Korea indicate that it can alleviate menopausal symptoms [47]. In Korea, hominis placenta is injected subcutaneously. It has been shown to improve menopausal symptoms and liver function.

The second problem involves blinding. In this study, the control group is administered NS, which differs in color and viscosity from PLC. Hence, medical doctors can easily identify the medication. Since this study is designed as a single-blind study, blinding of participants will be maintained by covering the syringe with opaque tape.

The third hurdle is that, due to the absence of previous studies, the power calculation was based on data from studies where hominis placenta was applied as an oral medication and from studies of acupuncture. The size of the control group was calculated based on the acupuncture studies, but we believe the NS injection in this study will be more effective than pure acupuncture due to the additional physical stimulation of the acupoints. This needs to be considered when analyzing the outcomes.

Lastly, there are various ways to evaluate hot flashes. We selected the hot flash score as the primary outcome evaluation, since it assesses both the frequency and intensity of hot flashes, enabling us to evaluate the efficacy of treatment more accurately.

This clinical trial is the first to use an objective basis to measure the efficacy and safety of hominis placenta pharmacopuncture. It is supported by national funding. We expect that this study will provide evidence for the efficacy and safety of hominis placenta pharmacopuncture. Based on the results, we plan to recommend a standard protocol for pharmacopuncture treatment and aim to substantiate clinical practice guidelines as a development of Korean medicine. We also expect hominis placenta pharmacopuncture to be used globally as an alternative therapy for hot flashes experienced by premenopausal and postmenopausal women.

### Trial status

Recruitment began on 31 October 2018. The current version of the protocol is 1.9, published on 1 November 2018. Recruitment is expected to be completed by the end of December 2019. The populated SPIRIT checklist is provided as Additional file 1.

## Additional file

Additional file 1: SPIRIT 2013 Checklist: Recommended items to address in a clinical trial protocol and related documents*. (PDF 129 kb)

## Data Availability

The datasets used or analyzed during the current study will be available from the corresponding author on reasonable request.

## Abbreviations

MRS: Menopause Rating Scale
NS: Normal saline
PLC: Hominis placenta extract

## References

[1] McKinlay SM, Jefferys M (1974). The menopausal syndrome. Br J PrevSoc Med.

[2] Shanafelt TD, Barton DL, Adjei AA, Loprinzi CL (2002). Pathophysiology and treatment of hot flashes. Mayo Clin Proc.

[3] Kronenberg F (1994). Hot flashes: phenomenology, quality of life, and search for treatment options. Exp Gerontol.

[4] Albertazzi P, Pansini F, Bonaccorsi G, Zanotti L, Forini E, De Aloysio D (1998). The effect of dietary soy supplementation on hot flushes. Obset Gynecol.

[5] Stearns V, Ullmer L, López JF, Smith Y, Isaacs C, Hayes D (2002). Hot flushes. Lancet.

[6] US FDA CDER. Estrogen and Estrogen/Progestin drug products to treat vasomotor symptoms and vulvar and vaginal atrophy symptoms-Recommendations for clinical evaluation. Guidance for Industry. https://www.fda.gov/downloads/drugs/guidancecomplianceregulatoryinformation/guidances/ucm071643.pdf. Accessed Jan 2003.

[7] Berendsen HH (2000). The role of serotonin in hot flushes. Maturitas..

[8] Chen CL, Weiss NS, Newcomb P, Barlow W, White E (2002). Hormone replacement therapy in relation to breast cancer. JAMA.

[9] Creasman WT (2002). Estrogen and cancer. Gynecol Oncol.

[10] Garg PP, Kerlikowske K, Subak L, Grady D (1998). Hormone replacement therapy and the risk of epithelial ovarian carcinoma; a meta-analysis. Obset Gynecol.

[11] Dong H, Ludicke F, Comte I, Campana A, Graff P, Bischof P (2001). An exploratory pilot study of acupuncture on the quality of life and reproductive hormone secretion in menopausal women. J Altern Complement Med.

[12] Wyon Y, Lindgren R, Lundeberg T, Hammar M (1995). Effects of acupuncture on climacteric vasomotor symptoms, quality of life, and urinary excretion of neuropeptides among postmenopausal women. Menopause..

[13] Portzio G, Trapasso T, Martelli S, Sallusti E, Piccone C, Mattei A (2002). Acupuncture in the treatment of menopause-related symptoms in women taking tamoxifen. Tumori..

[14] Huang NI, Nir Y, Chen B, Schnyer R, Manber R (2006). A randomized controlled pilot study of acupuncture for postmenopausal hot flashes: effect on nocturnal hot flashes and sleep quality. Fertil Steril.

[15] Hazel AP (2003). Hot flashes-A review of the literature on alternative and complementary treatment approaches. Altern Med Rev.

[16] Choi YN, Oh JY, Cho HS, Kim KH, Kim KS, Lee SD (2015). Research on the Amount of Stimulus Differences According to Pharma-copuncture Injected dose and Characters Method. J Acupunct Res.

[17] Park BK, Cho JH, Son CG (2009). Randomized Clinical Controlled Trials with Herbal Acupuncture (Pharmacopuncture) in Korea - A Systematic Review. J Korean Oriental Med.

[18] College of Oriental Medicine, Korea Herbalogy Professor (2000). Bonchohak.

[19] Lee SG, Lee JD, Koh HK, Park DS, Lee YH, Kang SK (2000). The Study on the Hominis Placenta Aqua-acupuncture Solution. J Acupunct Res.

[20] The Korean Pharmacopuncture Institute (1999). Pharmacopuncture intervention clinical guideline.

[21] Heo JK, Lee JM, Lee CH, Lee KS, Jang JB (2011). A Review of the Utility of Hominis Placenta on Oriental Obstetrics and Gynecology. J Orient Obstet Gynecol.

[22] Kim KM, Kim MJ, Hong SE (2003). Efficacy of Hominis Placenta Aqua-acupuncture Solution in the Treatment of Melasma. J Orient Med Ophthalmol Otolaryngol Dermatol.

[23] Yoo HS, Kang WC, Cho JH, Lee YW, Son CG, Cho CK (2005). Effects of Hominis Placenta Herbal Acupuncture (HPA) on Menstrual Cramps. J Pharmacopuncture..

[24] Chang SY, Kim HJ, Lee DY, Lee EY (2005). Effect of Hominis placenta herbal acupuncture on dysmenorrhea. Acupuncture.

[25] Kim SM, Jang SH, Kim CH, Yoon HM, Song CH, Ahn CB (2008). Effect of Hominis placenta pharmacopuncture on the dysmenorrhea a pilot study, single blind, randomized, controlled clinical trial. Aust J Pharm.

[26] Lee JH, Kim YH, Yook TH, Lee EY, Kim EH (2002). The Clinical Observation of peripheral facial paralysis used Aqua-acupuncture treatment. J Acupunct Res.

[27] Kim TH, Park KY, Park JE (2010). The effect of hominis placenta herbal acupuncture therapy on the postpartum women’s heat feeling, sweat and thirst. J Orient Obstet Gynecol.

[28] De D, Chakraborty PD, Bhattacharyya D (2011). Regulation of trypsin activity by peptide fraction of an aqueous extract of human placenta used as wound healer. J Cell Physiol.

[29] De D, Datta Chakraborty P, Mitra J, Sharma K, Mandal S, Das A (2013). Ubiquitin-like protein from human placental extract exhibits collagenase activity. PLoS One.

[30] Hong JW, Lee WJ, Hahn SB, Bj K, Lew DH (2010). The effect of human placenta extract in a wound healing model. Ann Plast Surg.

[31] Park JY, Lee J, Jeong M, Min S, Kim SY, Lee H (2014). Effect of Hominis Placenta on cutaneous wound healing in normal and diabetic mice. Nutr Res Pract.

[32] Yoon JS, Seo JC, Lee HS, Lim SC, Jung TY, Shin LH (2004). The Clinical Study on Acupuncture Sensation in Hwangryunhaedoktang Herbal Acupuncture and Hominis Placenta Herbal Acupuncture -The Basic Study on Placebo Herbal Acupuncture (2). J Acupunct Res.

[33] Finck G, Barton DL, Loprinzi CL, Quella SK, Sloan JA (1998). Definitions of Hot Flashes in Breast Cancer Survivors. J Pain Symptom Manag.

[34] Kim KH, Kang KW, Kim DI, Kim HJ, Yoon HM, Lee JM (2010). Effects of acupuncture on hot flashes in perimenopausal and postmenopausal women-a multicenter randomized clinical trial. Menopause..

[35] Zhong LL, Tong Y, Tang GW, Zhang ZJ, Choi WK, Cheng KL (2013). A randomized, double-blind, controlled trial of a Chinese herbal formula (Er-Xian decoction) for menopausal symptoms in Hong Kong perimenopausal women. Menopause..

[36] Xia Y, Zhao Y, Ren M, Zhang J, Wang Y, Chang Y (2012). A randomized double-blind placebo-controlled trial of a Chinese herbal medicine preparation (Jiawei Qing'e Fang) for hot flashes and quality of life in perimenopausal women. Menopause.

[37] Park Jimin, Lee Hyangsook, Shin Byung-Cheul, Lee Myeong Soo, Kim Boryang, Kim Jong-In (2016). Pharmacopuncture in Korea: A Systematic Review and Meta-Analysis of Randomized Controlled Trials. Evidence-Based Complementary and Alternative Medicine.

[38] Korean Pharmacopuncture Institute (2011). Pharmacopuncturology: Principles and clinical applications.

[39] Bernhard LA, Shappard L (1992). Health, symptoms, self-care and dyadic adjustment in menopausal women. J Obstet Gynecol Neonatal Nurs.

[40] Pansini F, Albertazzi P, Bonaccorsi G, Calisesi M, Campobasso C, Zanotti L (1994). The menopausal transition: a dynamic approach to the pathogenesis of neurovegetative complaints. Euro J Obstet Gynecol Reproduct Biol.

[41] Oldenhave A, Jaszmann L, Haspels A, Everaerd W (1993). Impact of climacteric on well-being. Am J Obstet Gynecol.

[42] Noh JH, Park JA, Yoon HM, Jang KJ, Song CH, Ahn CB (2009). The effect of hominis placenta pharmacopuncture on leg spasticity of stroke patients (a pilot study, double blind, randomized, controlled clinical trial). J Pharmacopuncture.

[43] Lee AR, Kim WI (2013). The Retrospective Comparative Study of General Acupuncture Therapy and Hominis placenta Pharmacopuncture Therapy on Severe Dyspepsia. Korean J Acupuncture.

[44] Lee CW, Kim HG, Heo SW, Jung KK, Ahn CB, Song CH (2005). The clinical study about hominis placenta herbal acupuncture on bell's palsy. Aust J Pharm.

[45] Park KB, Song KH, Lee JS, Jo JH (2006). Study on clinical effects of Hominis placenta herbal acupuncture on osteoarthritis of knee joint. J Korean Acupunct Moxibustion Soc.

[46] Eisenhardt S, Fleckenstein J (2016). Traditional Chinese medicine valuably augments therapeutic options in the treatment of climactreric syndrome. Arch Gynecol Obstet.

[47] Kim SM, Park HT, Lee BI, Shin JH, Park HM, Kin T (2013). Comparison of the efficacy and safety of the Unicenta and Melsmon injection for the menopausal symptoms. J Korean Soc Menopause.

